# Infective Endocarditis in Hypertrophic Obstructive Cardiomyopathy After Etonogestrel Implant Removal

**DOI:** 10.7759/cureus.29810

**Published:** 2022-10-01

**Authors:** Mohammad M Dlewati, Kamahl Harrisingh, Rannah Dabiri

**Affiliations:** 1 Internal Medicine, Memorial Healthcare System, Hollywood, USA; 2 Internal Medicine, Florida Atlantic University Charles E. Schmidt College of Medicine, Boca Raton, USA

**Keywords:** cerebral septic emboli, modified duke criteria, etonogestrel implant removal, etonogestrel implant, bacterial endocarditis, cardiovascular implantable-electronic devices, hypertrophic obstructive cardiomyopathy (hocm)

## Abstract

The modified Duke criterion "predisposing heart condition" is poorly defined, and is based on outdated studies of the epidemiology of infective endocarditis (IE). Hypertrophic obstructive cardiomyopathy (HOCM) is not classified as a modified Duke criterion for the diagnosis of IE but is associated with a higher incidence of IE nonetheless. The presence of a cardiovascular implantable electronic device (CIED) is independently associated with an increased risk of IE. Patients with HOCM may be candidates for the implantation of an automated internal cardiac defibrillator (AICD) for the prevention of sudden cardiac death. Previous studies of the risk of IE in patients with HOCM did not make a distinction for patients with CIEDs. We present a case of a 25-year-old female with HOCM and an AICD for primary prevention, who presented with sudden right-sided hemiplegia, aphasia, dysarthria, and a low-grade fever. CT angiography demonstrated large vessel occlusion of the terminal left internal carotid artery and proximal middle cerebral artery (MCA), prompting emergent treatment with mechanical thrombectomy, which achieved full recanalization and full reperfusion. Cardioembolic stroke was suspected. She had no arrhythmias, a transthoracic echocardiogram showed new mitral valve vegetation. The etiology of the stroke was determined to be septic emboli from mitral valve subacute bacterial endocarditis and two blood cultures grew staph epidermidis. Ten days prior to presentation, she had undergone removal of an etonogestrel implant in her arm, and this was the suspected source of initial bacteremia and valvular seeding. She was treated with a six-week course of vancomycin with improvement and maintained on daily minocycline as long as the AICD were to remain in place. Our patient started developing symptoms of endocarditis after the removal of her etonogestrel implant, had no other recent procedures, and had good dentition. Hence, we maintain that this was the likely source of her initial bacteremia that led to valvular seeding and resultant IE. This is the first reported case of etonogestrel implant removal-related endocarditis. Further studies of the association between etonogestrel implant removal, transient bacteremia, and valvular seeding leading to IE are warranted. Clinicians should be reminded of the increased risk of IE in patients with HOCM. Identifying HOCM patients at higher risk for IE, i.e. dilated left atrium and/or CIEDs is easier to accomplish with current cardiac imaging techniques.

## Introduction

Subacute bacterial endocarditis can be an elusive diagnosis that requires high clinical suspicion. Despite its limitations, the modified Duke criteria remain a commonly used clinical tool for the diagnosis of infective endocarditis (IE). One such limitation is in the poorly defined minor criterion “predisposing heart condition” [1]. The commonly described native heart conditions which are classified as predisposing heart conditions are mitral valve prolapse, prior IE, and bicuspid aortic valve. However, the exclusivity of this list is of questionable merit due to the advancements in knowledge, definitions, and diagnostics since the inception of the criteria [2-3]. Other conditions are known to be associated with a higher incidence of IE, however, are not included in the definition of “predisposing heart condition.” Among these are hypertrophic obstructive cardiomyopathy (HOCM) and the presence of cardiovascular implantable electronic devices (CIEDs) [4-6]. Etonogestrel implants are a form of long-acting reversible contraceptive typically implanted in the deep soft tissue of the arm. While the implantation is done with a pre-prepared specialized device and with minimal pain or damage to the patient, the removal of the implant is done surgically and can be complicated by fibrosis around the implant site, implant migration, or device fracture. In these cases, retrieval of the implant can involve extensive physical manipulation which could expose the site to infection and subsequently septicemia and endocarditis [7-8]. This is the first reported case of etonogestrel implant removal-related endocarditis.

## Case presentation

A 25-year-old female presented to the emergency department with a chief complaint of sudden onset weakness and speech difficulty. Her medical history was significant for HOCM and an implanted cardioverter-defibrillator (ICD) placement six years prior, for primary prevention due to a family history of sudden cardiac death. On presentation, she was noted to be hypotensive, tachycardic, tachypneic, and with an elevated temperature of 37.8°C. On physical exam, there was flaccid paralysis in the right upper and lower extremities, and moderate dysarthria with moderate expressive aphasia. Facial asymmetry was present with right-sided facial droop and no gaze preference. No erythema, swelling, or tenderness was appreciated in the chest wall over the site of the ICD generator. Labs on admission are displayed below in Table 1. 

**Table 1:** Admission labs including a basic metabolic panel, liver function panel, hemoglobinA1c, and complete blood count with differential. BUN, blood urea nitrogen; ALT, alanine transaminase; AST, aspartate transaminase; POCT, point-of-care testing; WBC, white blood cell; ANC, antineutrophil cytoplasmic antibodies; MCV, mean corpuscular volume; Abs, absolute; SGPT, serum glutamic pyruvic transaminase; SGOT, serum glutamic oxaloacetic transaminase

	Patient’s value	Reference range
BUN, Bld	10	7-18 mg/dL
Sodium	137	136-145 mmol/L
Potassium	3.9	3.5-5.1 mmol/L
Chloride	105	98-107 mmol/L
CO2	25	21-32 mmol/L
Anion gap	7	5-15 mmol/L
Calcium	8.6	8.5-10.1 mg/dL
Creatinine	0.73	0.51-0.95 mg/dL
Total protein	7	6.4-8.2 g/dL
Albumin	2.9 (L)	3.4-5.0 g/dL
Alkaline phosphatase	91	45-117 Units/L
ALT (SGPT)	28	13-56 Units/L
AST (SGOT)	16	13-37 Units/L
Bilirubin total	0.4	0.2-1.3 mg/dL
Glucose, POCT	92	70-99 mg/dL
Hemoglobin A1c	5.4	<5.7 %
WBC	19.5 (H)	3.5-10.0 1000/uL
ANC	16.80 (H)	2.00-7.15 10*3/uL
Red blood cell count	4.26	4.00-5.50 million/uL
Hemoglobin	14	11.4-15.4 g/dL
Hematocrit	41	32.8-45.6%
MCV	96.2 (L)	80.0-95.0 fL
Platelets	265	150-450 10*3//uL
Neutrophils	86.2 (H)	42.5%-73.2%
Lymphocytes	7.5 (L)	18.2%-47.4%
Neutrophils absolute	16.79 (H)	2.00-7.15 10*3/uL
Lymphocytes Abs	1.46	1.16-3.18 10*3/uL
Monocytes Abs	0.98 (H)	0.29-0.71 10*3/uL

**Figure 1:**
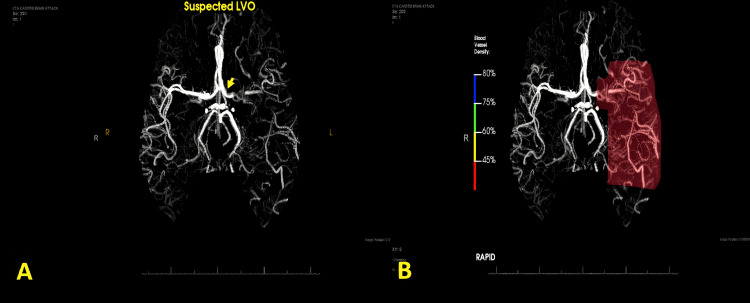
In image A on the left CT angiography reconstruction imaging reveals filling defects in the (yellow arrow) left terminal ICA, M1 and M2 segments of the MCA compatible with large vessel occlusion/thrombosis. In image B on the right, blood vessel density in left MCA territory was estimated by AI vessel density mapping technology to be severely reduced, greater than 55%. ICA, internal carotid artery; MCA, middle cerebral artery; AI, artificial intelligence

A CT of the brain without contrast (Figure 1) revealed no hemorrhage or other acute findings. Subsequent CT angiography of the brain and carotid arteries showed filling defects within the left terminal internal carotid artery and left M1 and M2 segments of the middle cerebral artery (MCA), compatible with acute thrombosis.

**Figure 2:**
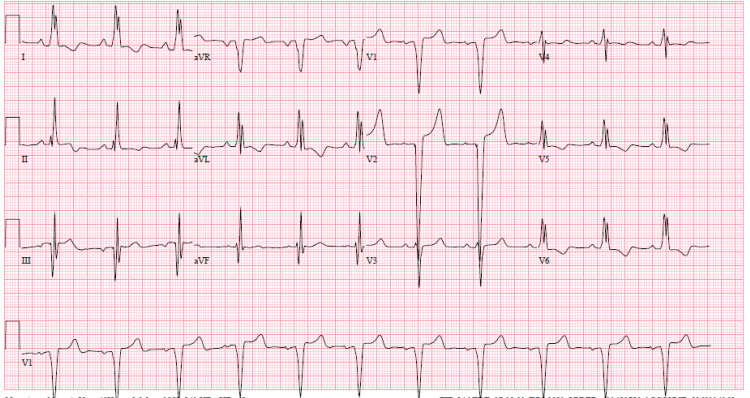
The EKG findings: normal sinus rhythm, left bundle branch block, ventricular rate 68 bpm, PR interval 176 ms, QRS duration 134 ms, QT/QTc 480/510 ms, P-R-T axes 9 29 169. EKG, electrocardiogram

**Figure 3:**
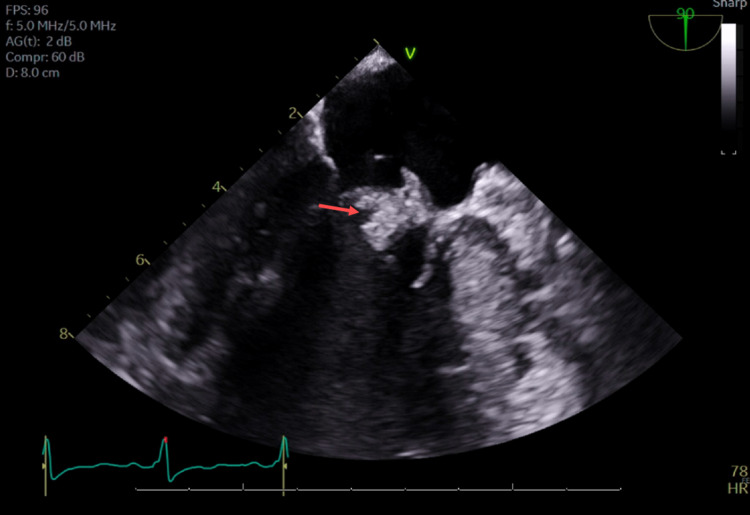
Transesophageal echocardiography visualizing the mitral valve vegetation (red arrow) measuring 1.5 cm x 0.9 cm attached to the anterior mitral leaflet on the ventricular side and prolapsing to the atrial side and left ventricular outflow tract. A moderate eccentric posterior jet of mitral regurgitation was noted. Other pertinent transesophageal echocardiographic findings included normal left ventricular systolic function with an estimated left ventricular ejection fraction of 65%-70%, significant left ventricular hypertrophy with systolic anterior motion of the anterior mitral leaflet, a defibrillator wire in the right atrium and right ventricle with no suspicious vegetation or thickening, normal tricuspid aortic valve, no evidence of intracardiac shunting with an agitated saline bubble study, and a clear left atrial appendage with no evidence of a thrombus.

The patient was taken emergently for angiography and possible mechanical thrombectomy. Digital subtraction angiography was performed showing left MCA M1 occlusion with absent perfusion (eTICI score 0). A mechanical thrombectomy was then performed with an aspiration catheter and stent retriever. After three passes, post-thrombectomy angiographic runs demonstrated full recanalization and full reperfusion of the left M1-M2 segment (eTICI 3). After the exclusion of a carotid artery dissection, the etiology of the patient's cerebrovascular ischemic event was suspected to be cardioembolic. A review of electrocardiograms (EKGs) (Figure 2), cardiac rhythm on telemetry, and ICD interrogation were unremarkable. Transthoracic echocardiogram was notable for severe LVOTO (LVOT gradient of 95 mmHg), severe left ventricular hypertrophy, and mitral valve thickening that was new compared to a prior echocardiogram -- although mitral valve visualization for characterization of the thickening was limited by the transthoracic approach (Figure 3). Therefore, the patient was started on empiric treatment with IV vancomycin and cefazolin. Blood cultures (Table 2) grew Staphylococcus epidermidis and swabbing of the nares confirmed colonization with methicillin-resistant *Staphylococcus aureus*. A transesophageal echocardiogram was then performed and confirmed a 1.5 cm x 0.9 cm vegetation attached to the anterior mitral leaflet and prolapsing to the atrial side. On further discussion, the patient denied any recent dental procedures or surgeries. She denied any illicit drug use. However, 10 days prior to admission the patient had an etonogestrel implant removed from her arm by an experienced provider after a 10-month period of implantation.

**Table 2:** Results of blood Gram stain, culture, and susceptibility testing.

Gram stain	Culture	Susceptibility
Gram positive cocci in clusters, two of two bottles of this set	*Staphylococcus epidermidis* (*Staphylococcus* species coagulase negative), two of two bottles of this set	Cefazolin: resistant clindamycin: sensitive minocycline: sensitive oxacillin: resistant rifampin: sensitive tetracycline: resistant trimethoprim/sulf: resistant vancomycin: sensitive

After consultation with a cardiac surgeon, no surgical intervention was done due to the patient's informed preference. The patient was continued on IV vancomycin therapy -- changed after four weeks to daptomycin for possible vancomycin-related infusion reactions -- and completed a total of six weeks. Transthoracic echocardiography after completion of the six weeks of parenteral antibiotic therapy showed marked improvement in the size of the vegetation and the patient's infection-related clinical signs and symptoms of fever, fatigue, chills, and tachycardia had resolved. In the presence of an AICD, the patient was maintained afterward on suppressive antibiotic therapy with oral minocycline 100 mg twice daily for as long as the cardiac device was to remain in place. The patient was seen for follow-up three months after completion of parenteral antibiotic therapy and was doing well with adherence to suppressive therapy and showed no signs of endocarditis recurrence. She was now using etonogestrel/ethinyl estradiol vaginal rings as a contraceptive. 

## Discussion

The modified Duke criteria are often used to help clinicians more accurately diagnose IE. In this case, the patient's presentation with a stroke, absence of a well-recognized predisposing heart condition as defined by the modified Duke criteria, and no recent dental procedures hindered an immediate consideration of IE. With the focus on acute stroke management, echocardiography was not prioritized and blood cultures were delayed. Without the findings from these tests, this patient initially met only two of the minor Duke criteria, purporting a "rejected endocarditis" classification. Not until the results of these diagnostics were available could we classify the patient as "possible" and then "definite" endocarditis. In the absence of a third minor criterion (i.e. pre-disposing heart condition), the patient did not meet the criteria for possible IE and this caused a delay in appropriate treatment. However, within the minor Duke criteria, predisposing heart condition remains poorly defined. What is commonly considered as pre-disposing conditions are previous cardiac valve surgery, previous IE, mitral valve prolapse with valve leakage, abnormal valves caused by rheumatic fever and degenerative conditions, and certain congenital heart disease [2]. HOCM being a genetic but not congenital disease is not included in any of these criteria.

Yet, there is a significant increased risk of IE in patients with HOCM. There is an estimated incidence of 3.8 per 1000 person-years in patients with HOCM (with left ventricular outflow tract obstruction defined as a maximal Doppler gradient ≥30 mmHg), and further increasing to 9.2 per 1000 person-years in patients with concurrent left atrial (LA) dilation (greater than or equal to 5.0 cm). There approximates to 130-306 times the incidence compared to that of 3-7 per 100000 person-years for non-IV drug abuse-related native valve endocarditis [9-10].

Our patient also had an ICD that further increased her risk for IE. The epidemiology of IE has significantly changed over the last decades, exemplified by the case of rheumatic heart disease which is now the predisposing condition in only a minority of patients who develop IE [11]. In contrast, CIED use around the world has had steady growth over the past three decades and is increasingly recognized as a risk factor for endocarditis. The incidence of pocket infection with bloodstream infection or device-related endocarditis has been found to be 1.14/1000 device-years (95% confidence interval, CI 0.47-2.74), and the risk with ICD is higher than that with a permanent pacemaker (PPM) [12]. The cumulative risk of endocarditis in patients with the combination of HOCM, left atrial (LA) dilation, and ICD has not been studied. 

Our patient started developing symptoms of endocarditis after the removal of her etonogestrel implant, had no other recent procedures, and had good dentition. Hence, we maintain that this was the likely source of her initial bacteremia that led to valvular seeding and resultant IE. There is reasonable evidence to conclude transient bacteremia occurs with surgery on skin that is not clinically infected [13-14]. In the absence of large, double-blinded, randomized controlled studies to determine the risk of surgical site infections of the skin and endocarditis, many of the current recommendations are based on available lower-level studies and a logical approach using risk assessment. Antibiotic prophylaxis is not recommended for non-dental procedures in the absence of active infection, with the class of recommendation 3 due to the low level of evidence [15-16]. Etonogestrel implant removal difficulties are well documented, with increasing difficulty noted when placement is done by a private or inexperienced practitioner. The most common adverse event reported with etonogestrel implant removal is encasement in fibrotic tissue, requiring multiple removal attempts. Local infections and skin reactions are rarely reported with insertion and even less often with removal [17-19]. No cases of complicating endocarditis or bacteremia have been described in the literature up to this point.

## Conclusions

This is the first reported case of etonogestrel implant removal-related endocarditis. Monitoring and reporting other cases of endocarditis or bacteremia associated with this procedure is warranted to better study the potential risk of systemic infection from etonogestrel implant removal. The HOCM is the most common inherited heart disease and carries an increased risk of IE than the general population. Patients may require CIED implantation for sudden cardiac death prevention and CIED increases the risk of IE. Patients with HOCM and LA dilation have a further increased risk of IE. Therefore, HOCM particularly with concurrent LA dilation and/or CIED presence should heighten the clinician’s suspicion of IE. Modified Duke criteria alone may mislead the clinician as HOCM with or without LA dilation and/or CIED is not included in the criteria. There is an opportunity for new research to describe the cumulative risk of IE in patients with HOCM, LA dilation, and CIED.
